# Analysis of strand-specific RNA-seq data using machine learning reveals the structures of transcription units in *Clostridium thermocellum*

**DOI:** 10.1093/nar/gkv177

**Published:** 2015-03-12

**Authors:** Wen-Chi Chou, Qin Ma, Shihui Yang, Sha Cao, Dawn M. Klingeman, Steven D. Brown, Ying Xu

**Affiliations:** 1 Computational Systems Biology Lab, Department of Biochemistry and Molecular Biology, and Institute of Bioinformatics, University of Georgia, GA 30602, USA; 2 BioEnergy Science Center, TN 37831, USA; 3 Biosciences Division, Oak Ridge National Laboratory, TN 37831, USA; 4 National Bioenergy Center, National Renewable Energy Laboratory, Golden, CO 80401, USA; 5 College of Computer Science and Technology and School of Public Health, Jilin University, Changchun, Jilin 130012, China

## Abstract

Identification of transcription units (TUs) encoded in a bacterial genome is essential to elucidation of transcriptional regulation of the organism. To gain a detailed understanding of the dynamically composed TU structures, we have used four strand-specific RNA-seq (ssRNA-seq) datasets collected under two experimental conditions to derive the genomic TU organization of *Clostridium thermocellum* using a machine-learning approach. Our method accurately predicted the genomic boundaries of individual TUs based on two sets of parameters measuring the RNA-seq expression patterns across the genome: expression-level continuity and variance. A total of 2590 distinct TUs are predicted based on the four RNA-seq datasets. Among the predicted TUs, 44% have multiple genes. We assessed our prediction method on an independent set of RNA-seq data with longer reads. The evaluation confirmed the high quality of the predicted TUs. Functional enrichment analyses on a selected subset of the predicted TUs revealed interesting biology. To demonstrate the generality of the prediction method, we have also applied the method to RNA-seq data collected on *Escherichia coli* and achieved high prediction accuracies. The TU prediction program named *SeqTU* is publicly available at https://code.google.com/p/seqtu/. We expect that the predicted TUs can serve as the baseline information for studying transcriptional and post-transcriptional regulation in *C. thermocellum* and other bacteria.

## INTRODUCTION

Derivation of all transcription units (TUs) expressed under designed conditions is essential to the elucidation of transcription regulation in bacteria as they are the basic functional units (1). A TU consists of a promoter, a transcriptional start site, a coding region containing one or multiple genes, and a transcription terminator (2). When a TU is expressed, its gene(s) are transcribed into a single RNA molecule (3,4). While the classical definition of operons is the same as that of TUs (3), a common practice in the past two decades, popularized by operon databases such as ODB (5), DBTBS (6), OperonDB (7) and DOOR (8,9), has been that operons do not overlap with each other and hence can be derived in general based on genomic sequence information alone. In contrast, a TU refers to a transcription unit that is dynamically composed by a specific triggering condition and different TUs may overlap with each other (10).

Because of their condition-dependent nature, hence difficult to predict computationally, there have not been many large datasets of TUs in the public domain for any organism before the emergence of the quantitative transcriptomic profiling technologies such as tiling arrays and RNA-seq. Hence functional studies of bacterial cells have to rely largely on operon predictions (11,12) or low throughput laborious experiments. Clearly, there is a fundamental difference between the predicted operons predicted based on sequence information only and the condition-dependent TUs, which makes operon-centric functional analyses, which are widely used in the literature, less informative for functional studies. As of now, a number of sets of TUs in *Escherichia coli* have been derived based on tiling array and RNA-seq data (1). A few large-scale RNA-seq datasets have also been generated on other bacteria for functional studies (13–15) as detailed in a recent review by Pinto *et al*. (16).

Some studies have been carried out to infer TUs through characterizing whole genome expression-patterns (14,17–18). For example, McClure *et al*. (17) and Fortino *et al*. (18) used predicted co-transcribed genes to evaluate their TU prediction. However, such studies suffer from without objective evaluation data against experimentally determined TUs. Thus, building a reliable and unbiased TU predictor remains an unsolved problem.

*Clostridium thermocellum* is a thermophilic bacterium and has been extensively studied in the recent past because of its potential as a consolidated bioprocessing organism for lignocellulosic ethanol production. In this study, we have analyzed four sets of ssRNA-seq data of *C. thermocellum* and predicted TUs using a machine-learning based method; and validated the prediction on a separate RNA-seq set, which has longer reads. This study provides a general method for accurate inference of TUs based on RNA-seq data collected under multiple conditions and can be used for TU prediction for other bacterial organisms when RNA-seq data are available.

## MATERIALS AND METHODS

### RNA sample and sequencing library preparation

*Clostridium thermocellum* ATCC27405 was grown on MTC medium in batch fermentation; and its transcriptomic levels have been measured previously using a DNA expression microarray (19). In that study, the treatment fermentations were exposed to 3.9 g/l (or 0.5% [v/v]) ethanol shock at a mid-exponential growth phase (Optical Density 600_nm_ ∼0.5) while the control fermentations were done without ethanol treatment. To derive TUs encoded in *C. thermocellum* ATCC27405, Illumina ssRNA-seq libraries were prepared from the total RNA extracted from one 60-min untreated control fermentation sample and one 60-min ethanol-shock sample obtained from the above study (19).

Our ssRNA-seq libraries were prepared according to an instruction manual provided by the manufacturer (Illumina, CA, USA), except that the poly-A selection step was omitted. Briefly, 300 ng of total RNA was fragmented for the directional RNA-seq libraries following by purification and addition of strand-specific adapters. Samples were then reverse-transcribed, amplified and DNA fragments were enriched by a final clean-up step according to the manufacturer instruction (Illumina). Libraries were normalized with duplex specific nuclease (Evrogen, Moscow, Russia) following the instruction in the DSN Normalization Application Note (Illumina). Final libraries were checked for quality control using an Agilent Bioanalyzer (Agilent, CA, USA) and quantified with a Qubit (Invitrogen, CA, USA). Libraries were then diluted and sequencing was done on version-1.5 single-read flow cell with TruSeq chemistry on an Illumina HiSeq 2000 instrument (20).

Four ssRNA-seq datasets were obtained from dilution of a 25 pM control library extracted from the control sample and dilutions of 25, 33 and 41 pM of the treatment library extracted from the treatment sample. The three concentrations of the same treatment library were designed to investigate how the concentrations and sequencing depths would influence the TU identification. Four datasets were obtained, named datasets 1–4 and used in our TU prediction. All the generated sequences have been deposited in the National Center for Biotechnology Information (NCBI) Sequence Read Archive (SRA) with accession number SRP002548.

In addition to the ssRNA-seq data outlined above, non-strand-specific RNA-seq datasets were also generated on the above four samples at multiple time points using the 454 GS FLX instrument (Roche, CA, USA), which produced reads with longer lengths, namely 225 bps for the average length versus 50 bps by Illumina sequencers. The 454 cDNA libraries were generated from the total RNA following the manufacturer's instructions, except that the small fragment removal step was not applied and the libraries were then sequenced using the Titanium chemistry (21). The 454 datasets were cleaned using SeqClean followed by mapping using the GSMapper program (Roche). The 454 datasets collected on biological replicates were combined.

### Quality check and characteristics of RNA-seq data

FastQC (http://www.bioinformatics.babraham.ac.uk/projects/fastqc/) was used to assess the read quality of the four ssRNA-seq datasets. All four datasets passed the check on both per base quality and per sequence quality (FastQC results were shown in Supplementary Figure S1 in the Supplementary Material). The reads were then mapped to the *C. thermocellum* ATCC 27405 genome (NC_009012.1) using Burrows-Wheeler Aligner (BWA) (22) with the default parameters. The mapping results were used to derive the following characteristics: (i) the average read depth, (ii) the antisense expression level and (iii) the coverage of coding regions and intergenic regions, respectively. Specifically, the average read depth is defined as:

\begin{equation*}
{\rm estimated}\;{\rm depth}(X) = \frac{{\sum\nolimits_{i \in G} {RAPSN(i)} }}{{|T|}}
\end{equation*}

where *G* and *T* represent the whole genome and the coding regions of *C. thermocellum*, respectively; |*T*| is the total length of sequence *T*; and *RAPSN*(*i*) denotes the read abundance at location *i* in the genome. Note that the estimated read depth does not consider intergenic regions.

### RNA-seq data of *E. coli*

Three *E. coli* RNA-seq datasets, generated with paired-end and strand-specific Illumina reads, were retrieved from the NCBI SRA database, with SRA accession numbers: SRX315217, SRX315218 and SRX315219, and used to test the generality of our TU prediction method. The three RNA-seq data were collected on triplicates of wild-type *E. coli K12 MG1655* that grew anaerobically in glucose minimal media (23). The generated RNA-seq data were mapped to the genome as single-end and paired-end reads, respectively, using BWA with the default parameters. The single-end mapping results were used to estimate the read coverage at the nucleotide level and the paired-end mapping results were used to determine the expressed intergenic regions and to predict TUs, where expressed intergenic regions refer to non-coding regions co-expressed with their flanking genes. The average lengths of the mapped paired-end reads including the genomic region between each paired-end, are 176, 160 and 174 bps for the three datasets.

### Training data preparation for TU prediction

Our TU predictors predict whether a consecutive gene pair on the same strand is co-transcribed into one single TU. An ideal training data would be RNA-seq data for experimentally verified TUs. However, currently there are no experimental data sufficiently large for training the TU predictors. Hence we took an alternative approach to prepare the training data. Specifically, for the negative training data, we have selected consecutive gene pairs deemed to be not in the same TU based on the following criteria: (i) the gap percentage out of the intergenic region between the two genes is >50%; and (ii) the ratio between the expression levels of the two genes is >10-fold. For the positive training data, we used 454 reads, which have longer reads than the ones by Illumina sequencers, to annotate consecutive gene pairs to be co-transcribed in the same TU based on the following criteria: (i) the intergenic region between a gene pair is fully covered by at least one 454 read; and (ii) the ratio between the expression levels of the two genes is less than and equal to two-fold. Although 454 reads provided some positive training data, they had a limitation in covering TUs whose intergenic regions are >225 bps. To have our positive training data cover TUs with longer intergenic regions, we added additional positive training data, as described in the following section.

### Generation of constructed TUs (cTUs)

We constructed a set of consecutive paired coding regions whose expression patterns along with those of the in-between regions resemble the expression patterns of true TUs, to provide additional training data. Each such paired coding region, along with the in-between intergenic region, is termed a constructed TU (cTU). We assume here that all defective RNA molecules due to the early release of the RNA polymerases from the DNA or truncation will be rapidly degraded by ribonucleases (24) and hence will not contribute to the observed expression levels. Therefore, an expressed TU should theoretically have a consistent expression level across a whole RNA molecule (10). However, due to technical reasons, the obtained RNA-seq data may not necessarily show such an expression-level consistency across an entire TU, which gives rise to fluctuations in the observed level across a TU.

To construct cTUs, we have used genes of *C. thermocellum*, whose coding regions each naturally fall into three sections, and resemble those of true TUs as defined in the previous section. To accomplish this, we plotted the length distribution of the annotated intergenic regions of *C. thermocellum*. Then, for each gene, we probabilistically selected a length for each made-up intergenic region according to this length distribution. Next, we assigned the made-up intergenic region within the gene spanning a region with the lowest GC content, knowing that true intergenic regions tend to have lower GC ratios (0.33) than protein-coding regions (GC ratio = 0.4) in *C. thermocellum* genome. The process of creating made-up intergenic regions is given as follows.

Denote the whole *C. thermocellum* genome (NC_009012.1) as *G*, which is 3 268 038 bps long and contains 1683 and 1680 genes on the forward and reverse strands, represented as *G*^+^ and *G*^−^, respectively, where $G^ + = \{ g_1^ + ,g_2^ + , \ldots g_{1,683}^ + \}$ and $G^ - = \{ g_1^ - ,g_2^ - , \ldots ,g_{1,680}^ - \}$; and the corresponding intergenic regions are represented as $IR^ + = \{ ir_1^ + ,ir_2^ + , \ldots ,ir_{1,682}^ + \}$ and $IR^ - = \{ ir_1^ - ,ir_2^ - , \ldots ,ir_{1,679}^ - \}$. We use the forward strand as an example to explain how made-up intergenic regions are created. The same procedure applies to the reverse strand.

***Step 1*:** Define *D*(*IR*^+^) as the density function of the $\frac{{|ir_i^ + |}}{{|ir_i^ + | + |g_i^ + | + |g_{i + 1}^ + |}}$ values and similarly define *D*(*GD*^+^) as the density function of the $\frac{{||g_i^ + | - |g_{i + 1}^ + ||}}{{|ir_i^ + | + |g_i^ + | + |g_{i + 1}^ + |}}$ values, for all consecutive gene pairs (25) in *G*^+^ without genes on the opposite strand in between.

***Step 2*:** For each gene $g_i^ + \in G^ +$, do the following to create a cTU through partitioning $g_i^ +$ into three regions, namely two coding regions and a made-up intergenic region (cIR):

***Step 2.1*:** select probabilistically *p_i_* and *q_i_* from *D*(*IR*^+^) and *D*(*GD*^+^), respectively, according to their density distributions. Set cIR in $g_i^ +$ to $p_i \times |g_i^ + |$.

***Step 2.2***: determine the start position of the cIR in $g_i^ +$ so that it satisfies,

\begin{equation*}
\mathop {\arg \min }\limits_{j \in [X,Y]} \{ g_i^ + [j,j + p_i \times |g_i^ + | - 1]_{GC} \} ,
\end{equation*}

where $X = m - \frac{{p_i \times |g_i^ + |}}{2} - \frac{{q_i \times |g_i^ + |}}{2}$ and $Y = m - \frac{{p_i \times |g_i^ + |}}{2} - \frac{{q_i \times |g_i^ + |}}{2}$ are the left and the right boundaries for the possible start position of the cIR in $g_i^ +$; *m* denotes the median position of $g_i^ +$; and $g_i^ + [a,b]_{GC}$ represents the GC level of the sub-region [*a, b*] of $g_i^ +$.

We then exclude those predicted cTU that violate the continuity and variance requirements of a TU, which are continuity and variance. For each predicted cTU, we remove it from further consideration if (i) the gap percentage of the intergenic region between the two genes is >50%; (ii) the ratio between the expression levels of the two genes is >10-folds; and (iii) plus the length of each predicted intergenic region being at least 225 bp long.

### Feature selection for TU predictors

We used two features extracted from gene-expression patterns, namely expression level ‘continuity’ and ‘variance’, originally proposed by Güell *et al*. (26) and Toledo-Arana *et al*. (27), both of which used a similar variance feature to detect TUs from RNA expression data generated using tiling arrays and predicted 139 and 517 polycistronic operons in *Mycoplasma pneumoniae* and *Listeria monocytogenes*, respectively. In addition, Oliver *et al*. also used continuous expressions over intergenic regions to detect 355 operons in *L. monocytogenes* based on non-strand-specific RNA-seq data (14).

For the ‘continuity’ feature, we used the following statistics to assess the expression gap in an intergenic region in a candidate cTU: a count of nucleotides with RAPSN = 0 in the region and the percentage out of the length of the region. For the *variance* feature, we used the following statistics to describe the variance of expression patterns across the two consecutive made-up coding regions in each candidate cTU: (i) fold change in expression levels between the consecutive genes and the intergenic region in between and (ii) the variance of the expression levels across the entire candidate cTU region. The cutoffs for these features are determined to give the best cross-validation results.

## RESULTS

### Characteristics of ssRNA-seq data

We examined the quality of ssRNA-seq data in terms of strand-specificity. The total expression levels on the incorrect (unexpected) strand were 0.55, 0.44, 0.44 and 0.44% of the total expression levels on the expected strand in the four ssRNA-seq datasets, respectively, where an unexpected strand was defined as an expressed DNA that appears on the opposite strand of an annotated gene.

Using the definition of whole-genome RAPSN (see ‘Materials and Methods’ section), we checked the read coverage by the ssRNA-seq data. Over 90% of all coding sequences (protein coding regions) were covered by at least one read. The sequence coverage of the intergenic regions varies from 22.06 to 30.72% across the four ssRNA-seq datasets. The estimated depths are listed in Table 1.

**Table 1.** Characteristics of *C. thermocellum* ssRNA-seq data

	Estimated depth (X)	Percentage of region covered by sequencing reads	
		Coding region	Intergenic region
dataset 1	480	91.77%	22.06%
dataset 2	935	95.85%	30.72%
dataset 3	576	94.35%	27.21%
dataset 4	305	91.83%	22.91%

### Training TU prediction model using only Illumina RNA-seq data

We first trained TU predictors using only cTU as the positive training data and non-TU gene pairs as the negative training data. Two groups of features were used in our training of the TU predictors: (i) expression-level continuity and (ii) expression-level variance. A support vector machine (SVM) toolkit, named libSVM (28), was used to train our binary classification models, with each predictor trained using a radial-basis function as the kernel function provided in libSVM. A five-fold cross-validation with the default parameters: ‘cost’, ‘gamma’ and ‘weight’, was employed. The four TU predictors achieved the accuracy levels at 0.93, 0.93, 0.92 and 0.93 for dataset 1–4 through the five-fold cross-validation, respectively; and the prediction sensitivities on the 792 and 663 TUs derived from the control and treatment sets of 454 data, respectively, at 0.9, 0.94, 0.94 and 0.92. The detailed receiver operating characteristic (ROC) curves are given in Supplementary Figure S7.

### Training TU prediction model using Illumina and 454 RNA-seq data

We used non-TU gene pairs as the negative training data and verified TUs based on 454 RNA-seq data plus the predicted cTUs as the positive training data for each ssRNA-seq dataset. The same features and training processes as the Illumina-only model were used to train this model. Overall, the four trained TU predictors achieved the accuracy levels at 0.94, 0.95, 0.95 and 0.95 on the four training sets, respectively. The ROC curves for the predictions are presented in Supplementary Figure S8.

### TU prediction in *C. thermocellum* by trained predictors

**Figure 1.**
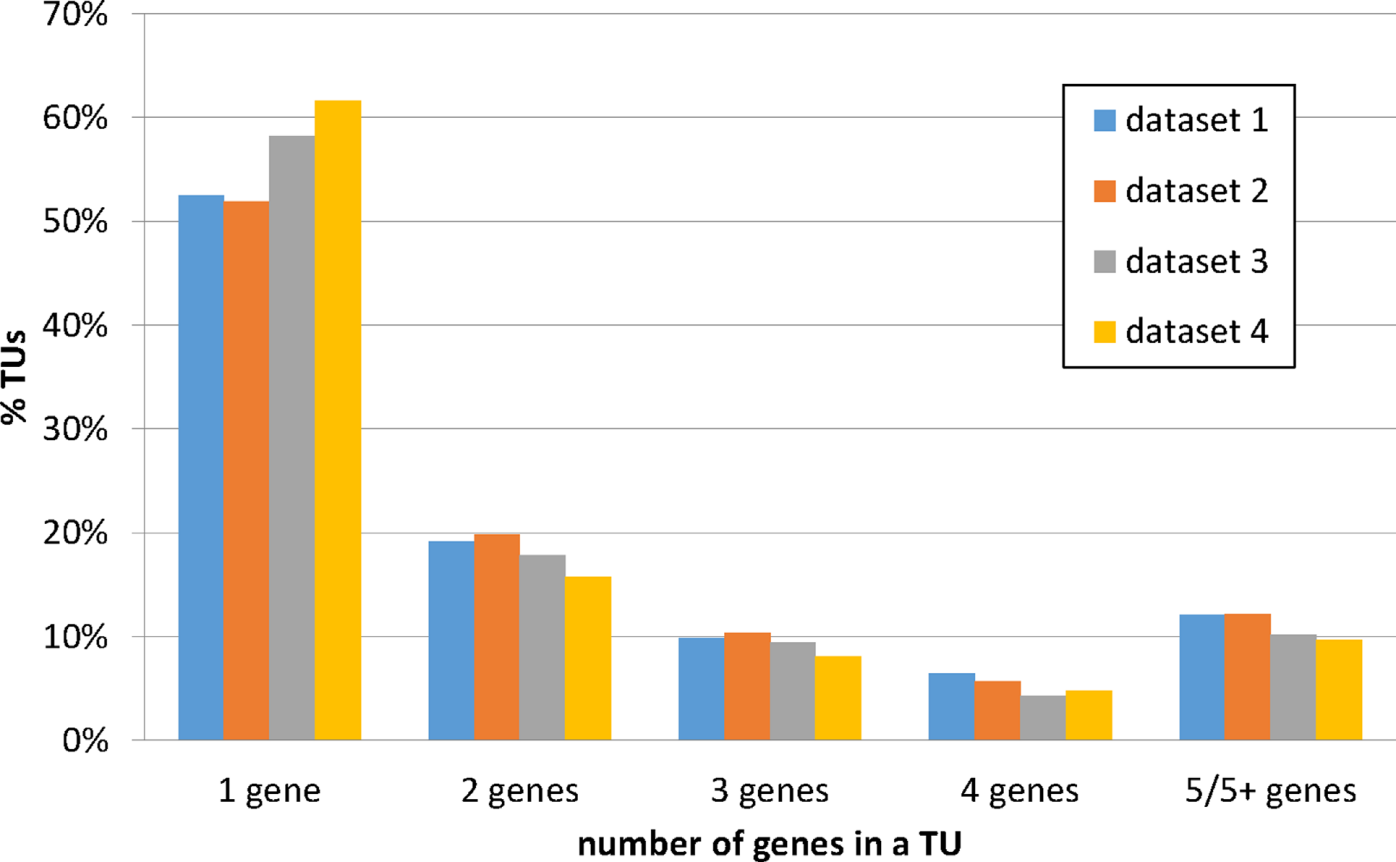
TUs predicted in *Clostridium thermocellum* stratified by the number of genes. The x-axis denotes the categories of TUs containing different numbers of genes and the y-axis denotes the percentage of TUs in each category.

A total of 2590 distinct TUs were predicted, with 1402, 1398, 1545 and 1604 TUs in datasets 1 through 4, respectively. The detailed TUs were shown in Supplementary file 1. Figure 1 shows the percentages of TUs having different numbers of genes. On average, 56% of TUs each contain one gene and 44% of TUs each have multiple genes, one-fourth of which have five or more genes. Dataset 2 has the lowest percentage of single-gene TUs and the highest percentage of multiple-gene TUs with five or more genes. The datasets 2 through 4 were sequenced using the same library, which together consist of 2238 distinct TUs. Among these TUs, 874 (39%) TUs were identical. The longest TU has 37 869 bps predicted in dataset 2, consisting of three consecutive and non-overlapping genes (Cthe_0041, Cthe0044 and Cthe0057). The TU containing the most number of genes, a total of 38, was 3477 bp long in dataset 2. Of the 38 genes, 19 encode flagellar related proteins (Cthe_0462-Cthe0485).

We observed that TUs on the leading-strand have more genes than TUs on the lagging-strand. This is consistent with a previous publication, which found that the leading strands tend to have more multiple-gene TUs than the lagging strands in ∼200 bacterial genomes (29). We examined the distributions of the predicted TUs on the leading versus the lagging strands. Using a published method (30), we noted that *C. thermocellum* has 2552 genes on the leading strand and 637 on the lagging strand. In addition, multi-gene TUs account for 81% of all TUs on the leading strand while 19% on the lagging strand. The average gene count per TU is 2.6 on the leading strand and 1.5 on the lagging strand. The detailed results are shown in Supplementary Figure S2.

### Evaluation of the predicted TUs

We have assessed the accuracy level of the predicted TUs, based on the confirmed TUs by the 454 RNA-seq datasets, where a TU is considered to be confirmed if it is entirely covered by at least one 454 read. Specifically, the prediction sensitivities of the four TU predictors are at 0.90, 0.94, 0.94 and 0.93, respectively, when 396 and 331 confirmed TUs in the control and the treatment datasets are compared against the predicted TUs in the corresponding datasets. We showed a discordant case in Supplementary Figure S3 to explain why our predictions cannot identify TUs suggested by 454 data.

We also assessed the reliability of the predicted TUs using a few general genomic features along with transcriptomic data as follows since there are no other large-scale experimentally verified TUs in *C. thermocellum*: (i) enrichment of *cis*-regulatory motifs in the immediate upstream regions of the predicted TUs (31,32); (ii) occurrences of the (predicted) transcription terminators in the immediate downstream regions of the predicted TUs; and (iii) the dominant expression level of inside-TU intergenic regions compared to outside-TU intergenic regions. We observed that (i) the promoters of the predicted TUs are statistically enriched with *cis*-regulatory motifs measured using nine documented motifs in *C. thermocellum* collected from RegTransBase (33), whose details can be found in Supplementary Method S1 (34,35) and Supplementary Figure S4; (ii) the immediate downstream regions of the predicted TUs are enriched with transcriptional terminators, as shown in Supplementary Method S2 (36) and Supplementary Figure S5; and (iii) inside-TU intergenic regions have significantly higher expression levels than outside-TU intergenic regions (Supplementary Method S3 and Figure S6). All these data suggest that the overall reliability of our prediction is high.

### Relationships among sequencing depth, predicted numbers of TUs and TU prediction accuracy

**Figure 2.**
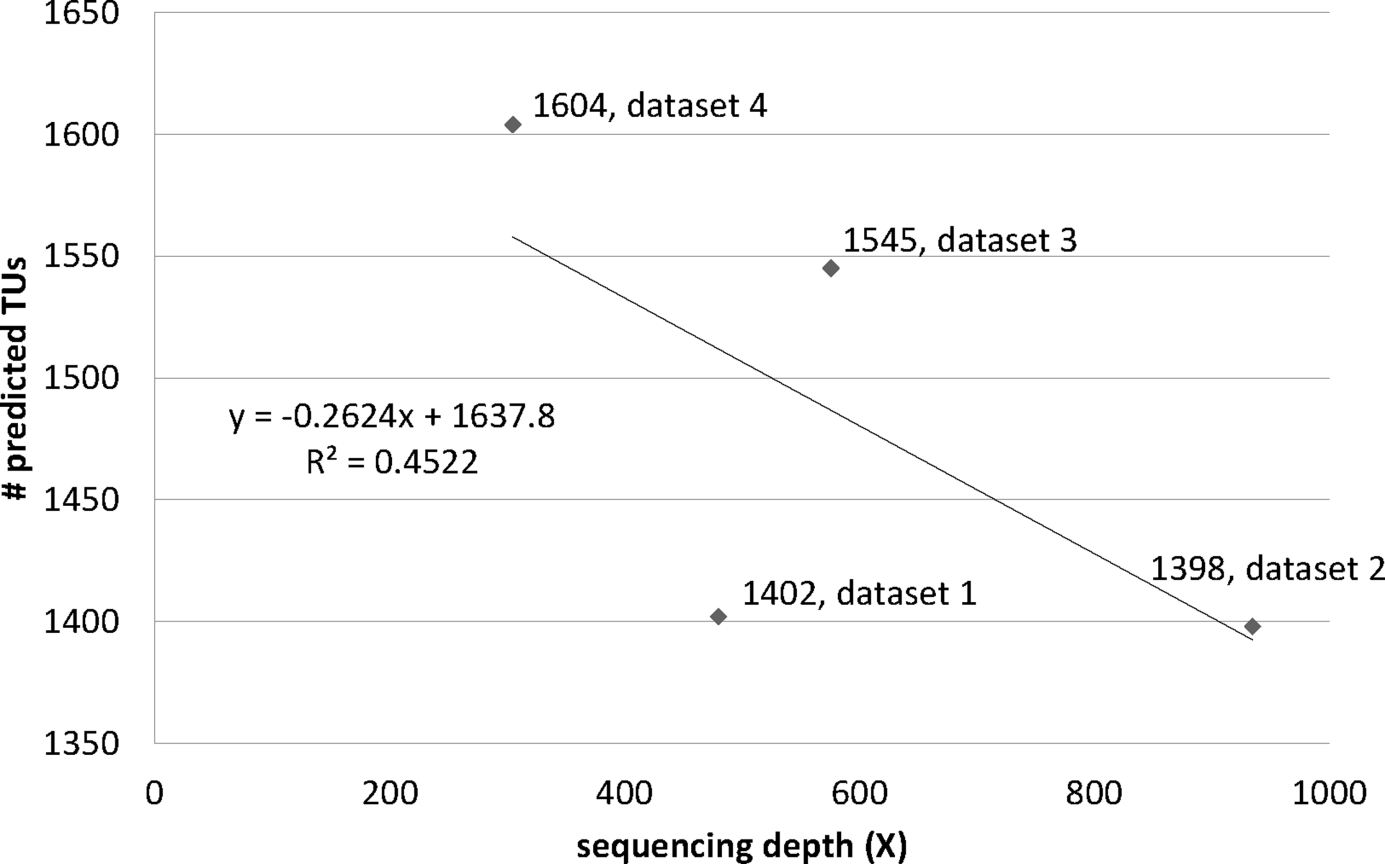
Relationship between sequencing depth and the number of predicted TUs.

The relationship between sequencing depth and the number of predicted TUs is shown in Figure 2. A regression line exhibits that the number of predicted TUs decreases as the sequencing depth increases. Dataset 2 had the highest sequencing depth (935X), the least number of predicted TUs and the most number of multiple-gene TUs, suggesting that higher sequencing depth may help to detect lowly-expressed genes and intergenic regions and hence provide information for connecting lowly-expressed genes and intergenic regions into TUs and result in lower numbers of TUs and larger numbers of multiple-gene TUs. We did note that TUs consisting of lowly expressed genes tend to have lower TU-prediction performance (results shown in Supplementary Table S1 in the Supplementary Materials).

**Figure 3.**
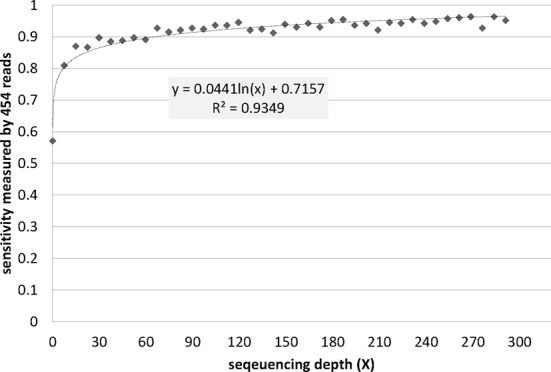
Relationship between sequencing depth and the TU prediction accuracy.

To further assess the stability of our method when applied to RNA-seq data with different sequencing depths, we have examined the relationship between the sequencing depth and the performance of TU prediction. We used dataset 4 as an example and performed reads resampling (without replacement) to simulate datasets with 40 different levels of sequencing depths ranging from 0.1X to 291X, with an increment of 7.46X. To avoid resampling bias, we performed 25 resampling experiments for each depth. The TU prediction results of resampling experiments were evaluated by the 454 dataset using the same process mentioned in the last section. Figure 3 shows that the prediction sensitivities are at 0.9 with sequencing depth at least 60X and decrease dramatically to 0.57 when the sequencing depth is 0.1X.

### Functional enrichment of the differentially composed TUs

To elucidate the dynamic nature of TUs under different conditions of ethanol treatment, we performed functional enrichment analysis of differently composed TUs extracted from 1402 and 1604 TUs in dataset 1 (control condition) and dataset 4 (treatment condition), respectively. They share 1007 TUs and dataset 4 consists of 597 differently, where 341 shared the same 5′ genes and 341 shared the same 3′ genes with those in the control set. Among the 597 TUs, 88 have their genes differentially expressed in dataset 4 compared to dataset 1 (with fold change cutoff as 4), which consist of 265 genes; and such genes are considered to be relevant to cellular responses to ethanol treatment. The longest TU among the 88 contains 14 genes (Cthe_2470–2482), which are partially related DNA adenine methylase. The corresponding TU under the control condition consists of the first ten genes and the other four fall into a separate TU.

Our function-enrichment analysis of these genes using DAVID (37) revealed that electron transport is the most enriched biological functions by the 88 TUs covering 265 genes (see detailed list in Supplementary file 2). The analyses revealed that 18 of the 265 genes enriched the electron transport. This function was confirmed by a previous study where genes related to it were differentially regulated under the ethanol stress condition, to overcome energy shortage caused by inhibition of glycolysis (19). The other most enriched pathways/functional categories include glutamate synthase and cell envelop. Our detailed enrichment data are given in Supplementary file 2. Further investigation of these differentially arranged TUs could potentially reveal more insights into gene function and regulation relevant to ethanol stress and responses.

### Application of the TU predictors to *E. coli* RNA-seq data

We used three sets of RNA-seq data of *E. coli K12 MG1655* to check the generality of our TU predictors. Specifically, we have built TU predictors based on *E. coli* data (single-end reads) as done on *C. thermocellum* data in Illumina-only model. The three trained TU predictors achieved the training accuracy at 0.95, 0.94 and 0.93 on datasets SRX315217, SRX315218 and SRX315219, respectively, with the ROC curves given in Supplementary Figure S9. 1003, 1340 and 1095 annotated TUs based on corresponding paired-end are used to assess the prediction sensitivities on the three datasets, which achieve a sensitivity level at 0.8, 0.9 and 0.92, respectively.

## DISCUSSION

In this study, we developed a computational method to predict bacterial TUs using RNA-seq data and two features of expression patterns across two consecutive genes and their intergenic region. A total of 2509 distinct TUs were predicted in *C. thermocellum* and evaluated using a few general genomic features along with a reliable transcriptomic dataset. We observed an association between sequencing depth and the number of predicted TUs, which reveals that a good level of TU prediction requires at least 60X sequencing depth. In addition, the read resampling experiments have shown that TU prediction performance is stable with sequencing depths beyond 60X, while it goes down dramatically when the sequencing depth is below 7.6X. The result suggests 7.6X as the minimum sequencing depth for reliable TU prediction using our method.

Lack of large-scale, experimentally verified TUs as training data is a bottleneck in developing computational models for TU identification and evaluation of the prediction results. Some TU prediction studies used mostly predicted operons to evaluate their TU annotations, which is clearly not appropriate knowing the fundamental difference between operons and TUs. Some studies also found new, alternative, extended operons (or expressed transcription units) not identified by operon predictions (38,39). Our TU prediction model was designed to identify TUs according to available RNA-seq data to infer the condition-dependent TU structures. To examine the level of difference between our TU prediction and currently available operons, we have defined a similarity score between a pair of TU sets in terms of the level of agreement between consecutive gene pairs belonging to the same TUs in the two sets (see Supplementary Method S4) and calculated the similarity scores on predicted TUs and operons in DOOR datasets. The similarity scores range from 0.53 to 0.54, as shown in Supplementary Table S2. As a comparison, the average similarity scores among the four TU sets are over 0.76 (from 0.71 to 0.82); furthermore the scores among datasets 2–4, with the same treatment but different concentrations, have an average level at 0.8.

We fully expect that more transcriptomic data will lead to improved prediction of TUs. For example, 5′ enriched sequencing data should help to improve the prediction of transcription start sites. In addition, more informative RNA-seq libraries such as paired-end reads with longer in-between regions may reveal better continuity information of a transcript. Another area for improvement, with more challenging issues, is to predict overlapping TUs expressed under the same conditions, which we plan to do in the near future.

## SUPPLEMENTARY DATA

Supplementary Data are available at NAR Online.

SUPPLEMENTARY DATA
