# Supplementary material for: Analysis of strand-specific RNA-seq data using machine learning reveals the structures of transcription units in Clostridium thermocellum
Source: Nucleic Acids Res. 2015 Mar 12;43(10):e67. doi: 10.1093/nar/gkv177 (PMC4446414; doi:10.1093/nar/gkv177)
Supplement: SUPPLEMENTARY DATA [file supp_43_10_e67__index.html]

Analysis of strand-specific RNA-seq data using machine learning reveals the structures of transcription units in Clostridium thermocellum — Analysis of strand-specific RNA-seq data using machine learning reveals the structures of transcription units in Clostridium thermocellum — SUPPLEMENTARY DATA 

# Analysis of strand-specific RNA-seq data using machine learning reveals the structures of transcription units in *Clostridium thermocellum*

## SUPPLEMENTARY DATA

**Files in this Data Supplement:**

- SUPPLEMENTARY DATA
- SUPPLEMENTARY DATA
- SUPPLEMENTARY DATA
