# Supplementary material for: Analysis of strand-specific RNA-seq data using machine learning reveals the structures of transcription units in Clostridium thermocellum
Source: Nucleic Acids Res. 2015 Mar 12;43(10):e67. doi: 10.1093/nar/gkv177 (PMC4446414; doi:10.1093/nar/gkv177)
Supplement: SUPPLEMENTARY DATA [file supp_gkv177_nar-03206-met-n-2014-File005.docx]

**Supplementary Materials of**

**Analysis of strand-specific RNA-seq data using machine learning reveals the structures of transcription units in** ***Clostridium thermocellum***

| a. Dataset1 | b. Dataset2 |
| --- | --- |
| 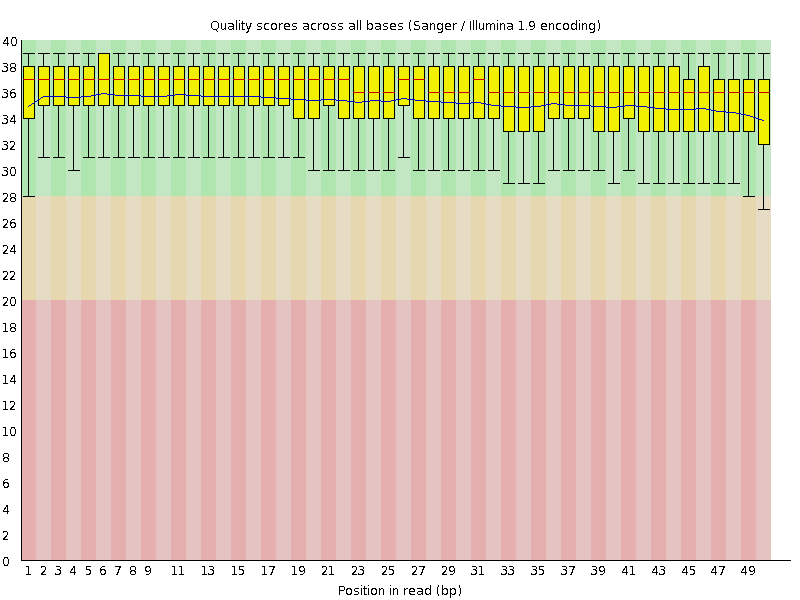 | 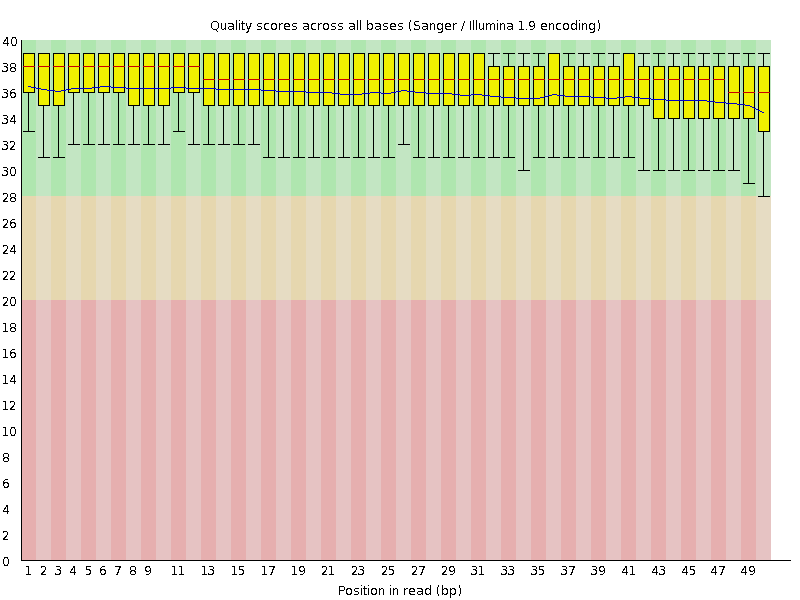 |
| c. Dataset3 | **d. Dataset4** |
| 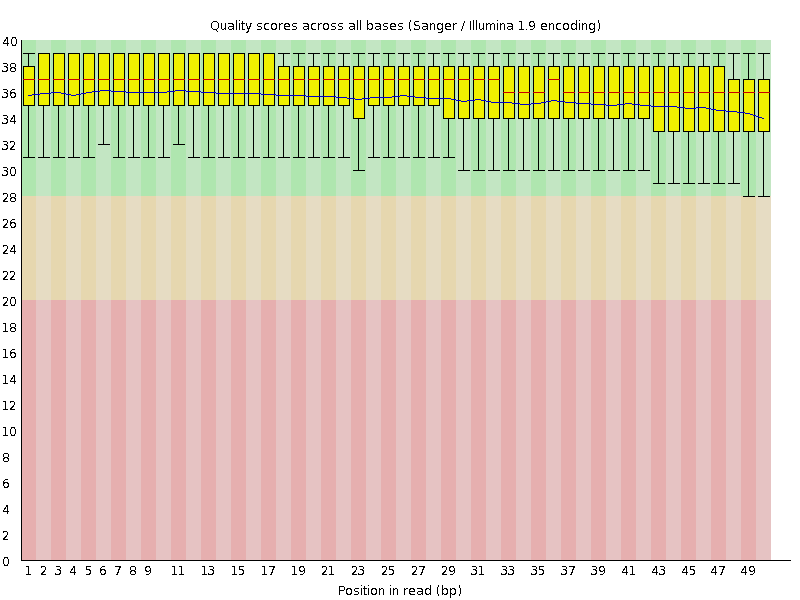 | 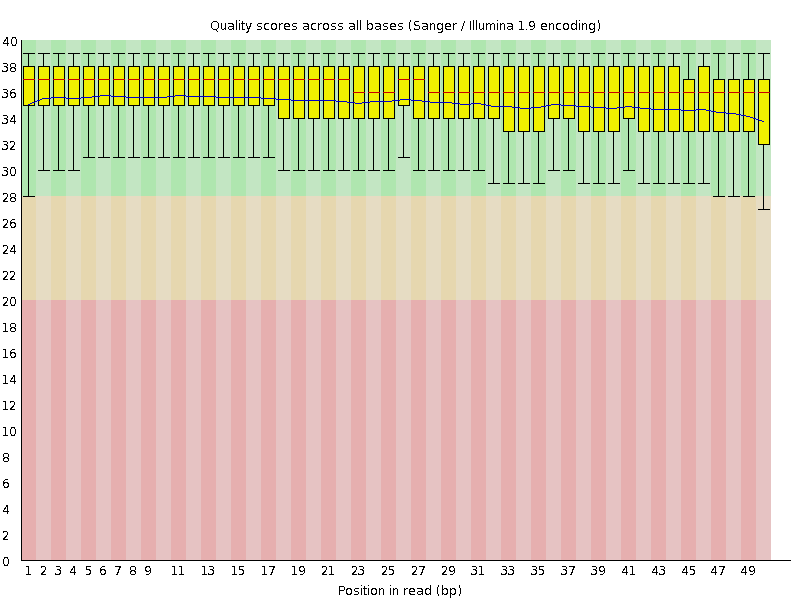 |

**Figure S1:** FastQC results for per base quality on the four datasets.

**Figure S2:** The percentages of TUs having different numbers of genes on the leading and lagging strands. The x-axis denotes the TUs containing different numbers of genes on the two strands, and the y-axis denotes the percentage of TUs in each ssRNA-seq dataset.

**Figure S3: Illumina RNA-seq signals of dataset 1 to 4 and 454 RNA-seq data of control and treatment over two forward genes, Cthe3321 and Cthe0631.**

Both 454 RNA-seq data (in bottom plot, black line is control and green line is treatment) supported that the genes Cthe_3321 and Cthe_0632 were co-transcribed into a TU. However, the four illumina RNA-seq datasets showed different signal patterns with very high fold changes of expression levels between the two genes. Because of the high fold changes, our TU prediction models determined that the two genes were not co-transcribed into the same TU. Although our TU prediction models can identify over 90% of TUs suggested by 454 data, we can still find a few cases showing the different RNA-seq signal patterns across different sequencing platform.

**Method S1: Analysis of *cis*-regulatory motifs regarding our TU prediction**

After using BBS (1,2) to scan all motifs, we designed an enrichment score to measure the difference on occurrences in the 5’ regions of identified TUs *versus* randomly selected genes, where we can get a positive enrichment score if the motifs are more enriched in the promoters of identified TUs than in those of the randomly picked genes. The enrichment score was designed as ${log}_{2}\left( \frac{\frac{N_{P}}{\left| P \right|}}{\frac{N_{R}}{\left| R \right|}} \right),$where |X| denotes the number of elements in X; N_P_ and N_R_ represent the numbers of motif instances in P and R, respectively. Here P represented the promoters of the identified TUs and R represents a set of randomly selected intergenic regions with |R|=|P|. We performed 1,000 rounds of enrichment-score calculations using 1,000 permutations of the 80-bp promoter regions. The promoter of a gene is defined as the 60-bp upstream and 20-bp downstream of the transcription start site (TSS), which was the first expressed position upstream of the identified TU (3).

If our TU identification is correct, we would expect to see enriched occurrences of *cis*-regulatory motifs at the 5' regions of the TUs compared to randomly selected 5' regions of genes. We used all the nine documented *cis*-regulatory motifs in *C. thermocellum* collected from RegTransBase (4) to check against our identification. The enrichment scores of the four Illumina RNA-seq data sets indicate that the annotated motifs were more enriched in the promoters of identified TUs than in those of the randomly picked genes, shown in Figure S4.

**Figure S4:** Enrichment scores of identified motifs in the promoter regions of the predicted TUs compared to those of randomly selected genes. The enrichment score should be more than 0 if the motifs are more enriched in the promoters of identified TUs than in those of randomly picked genes.

**Method S2: Rho-independent transcription terminators appear more frequently in common boundaries of predicted TUs than in unique boundaries**

The rho-independent transcription terminator is one of the two major classes of terminators and the only type that is computationally predictable by the state-of-the-art tools. We checked the occurrences of such terminators, predicted by TransTermHP (5), in the 3’ region at the end of each predicted TU. The following ratio was designed to measure the quality of the predicted TUs:

$$ratio=\frac{T}{NT}$$

where *T* represented the number of intergenic regions with at least one predicted terminator and *NT* represented the number of intergenic regions without any predicted terminator. This ratio for all intergenic regions in *C. thermocellum* was 0.31 without considering any TUs in its genome. We further partitioned all these intergenic regions into five categories based on the number of ssRNA-seq datasets supporting that an intergenic region located at an inter-TU region (shown in x-axis of Figure S3). For example, an intergenic region was classified into category 2 when two out of the four datasets suggested that the region located at inter-TU regions; and an intergenic region in category 4 was suggested by all the four data sets. Our result showed that the more common inter-TU regions tended to have higher ratio of terminator appearance as shown in Figure S5.

**Figure S5**: Rho-independent transcription terminators appear more frequently in the common boundaries of our identified TUs (categories 2, 3, and 4). The x-axis represents different level of common inter-TU regions. Y-axis denotes the terminator appearance ratio.

**Method S3: Intergenic regions inside TUs have higher expression levels**

If a TU is correctly identified, we expect that the intergenic regions within the TU should have higher expression levels compared to the inside-TU regions. Figure S6 shows the observed expression levels for intergenic regions inside *versus* outside of TUs. A Wilcoxon two-sample test showed that the statistical significance of the observed differences in expression levels inside *versus* outside of TUs are 5.5 × 10^-103^, 2.3×10^-74^, 1.1×^-100^, and 8.5×10^-120^, respectively, for datasets 1 through 4, significantly showing expression differences between inside- versus outside-TU regions.

**Figure S6:** The different expression levels of the intergenic regions between inside- and outside-TUs. The x-axis denotes the four datasets used, and the y-axis denotes the log_2_-transormed expression levels of the intergenic regions.

**Table S1:** TU prediction accuracy in different TU expression levels.

| **TU expression level range** | **# TU evaluated in the range  of expression levels** | **sensitivity** |
| --- | --- | --- |
| [0,20] | 31 | 0.58 |
| (20,70] | 82 | 0.95 |
| (70,120] | 46 | 1.00 |
| (120,170] | 36 | 1.00 |
| (170,220] | 31 | 0.97 |
| (220,270] | 15 | 1.00 |
| (270,320] | 14 | 1.00 |
| (320,370] | 11 | 1.00 |
| (370,420] | 9 | 1.00 |
| (420,470] | 8 | 1.00 |
| (470,520] | 6 | 1.00 |
| (520,570] | 2 | 1.00 |
| (570,7.01e+03] | 40 | 0.85 |

We evaluated TU prediction accuracy using TUs suggested by 454 RNA-seq data, and we used the expression level of TU to check the relationship between expression level and TU prediction accuracy. In dataset4, we got the prediction sensitivity from sets of TUs grouped by their expression levels. The overall prediction sensitivity in dataset 4 is 0.93. Our results (shown in the table) showed that the group with the lowest expression level had the lowest sensitivity (0.58), and the group with the highest expression level had lower sensitivity.

**Method S4: Difference between our identified TUs and the operons in DOOR**

DOOR database (6) represents one of the highest-quality operon predictions (7). According to DOOR's results, 56.07% of genes in *C. thermocellum* are in polycistronic operons while our results show that percentage of TUs containing multiple genes is in the range between 68.83% and 77.12% under the condition we used. To systematically compare our identified TUs with operons in DOOR, we have constructed transcriptional profiles for each TU set under four conditions as well as for the operon set from DOOR as follows. Let {*g_1_, g_2_,…, g_n_*} be the list of *n* genes encoded in the genome, arranged in the increasing order of their distance to the start of the genome. A *transcriptional profile* (TP) for a TU or operon set is defined as *C = {c_1_, c_2_,…, c_n_}*, with *c_i_= 1* if *g_i_* and *g_i+1_* are in the same TU (or operon), otherwise *c_i_= 0*; and *g_n+1_* = *g_1_*.

**Table S2:** The Jaccard similarity coefficient for each pair of transcriptional profiles

|  | DOOR | dataset 1 | dataset 2 | dataset 3 | dataset 4 |
| --- | --- | --- | --- | --- | --- |
| DOOR |  |  |  |  |  |
| dataset 1 | 0.5372 |  |  |  |  |
| dataset 2 | 0.5368 | 0.7481 |  |  |  |
| dataset 3 | 0.5335 | 0.7109 | 0.7967 |  |  |
| dataset 4 | 0.5402 | 0.7118 | 0.7716 | 0.8240 |  |

The *Jaccard similarity coefficient* (JC) between two TP sets is calculated as the size of the intersection between the two profiles divided by the size of their union, to measure the consistency level between two TUs (or between TU and operon sets). Note that the larger a JC value is, the higher the consistency level is between two TPs. As shown in Table S2, the JC values between the predicted TU sets and operon set are not high in general, with 0.5402 being the highest. As a comparison, the average pair-wise JC values among the four TU sets are over 0.76; furthermore the JC values among dataset 2-4, with same treatments but different concentrations, have a higher average level, 0.7974, which consistent with the experimental design.

**Figure S7:** The ROC curves of training TU prediction model using only Illumina RNA-seq data in *C. thermocellum*. ROC curves were performed with 5-fold cross-validation and optimal parameters cost, gamma and weight of libSVM package. X-axis is false positive rate and Y-axis is true positive rate. Pink, green, blue, and purple lines indicated the ROC curves of dataset1, dataset2, dataset3, and dataset4.

**Figure S8:** The ROC curves of training TU prediction model using Illumina and 454 RNA-seq data in *C. thermocellum*. ROC curves were performed with 5-fold cross-validation and optimal parameters cost, gamma and weight of libSVM package. X-axis is false positive rate and Y-axis is true positive rate. Pink, green, blue, and purple lines indicated the ROC curves of dataset1, dataset2, dataset3, and dataset4.

**Figure S9:** The ROC curves of training TU prediction model using only Illumina RNA-seq data in *E. coli*. ROC curves were performed with 5-fold cross-validation and optimal parameters cost, gamma and weight of libSVM package. X-axis is false positive rate and Y-axis is true positive rate. Pink, green, and blue lines indicated the ROC curves of SRX315217, SRX315218, and SRX315219.

**REFERENCES**

1. Li, G., Liu, B., Ma, Q. and Xu, Y. (2011) A new framework for identifying cis-regulatory motifs in prokaryotes. *Nucleic acids research*, **39**, e42.

2. Ma, Q., Liu, B., Zhou, C., Yin, Y., Li, G. and Xu, Y. (2013) An integrated toolkit for accurate prediction and analysis of cis regulatory motifs at a genome scale *Bioinformatics*.

3. Salgado, H., Peralta-Gil, M., Gama-Castro, S., Santos-Zavaleta, A., Muniz-Rascado, L., Garcia-Sotelo, J.S., Weiss, V., Solano-Lira, H., Martinez-Flores, I., Medina-Rivera, A. *et al.* (2013) RegulonDB v8.0: omics data sets, evolutionary conservation, regulatory phrases, cross-validated gold standards and more. *Nucleic acids research*, **41**, D203-213.

4. Cipriano, M.J., Novichkov, P.N., Kazakov, A.E., Rodionov, D.A., Arkin, A.P., Gelfand, M.S. and Dubchak, I. (2013) RegTransBase - a database of regulatory sequences and interactions based on literature: a resource for investigating transcriptional regulation in prokaryotes. *BMC Genomics*, **14**, 213.

5. Kingsford, C.L., Ayanbule, K. and Salzberg, S.L. (2007) Rapid, accurate, computational discovery of Rho-independent transcription terminators illuminates their relationship to DNA uptake. *Genome Biol*, **8**, R22.

6. Mao, F., Dam, P., Chou, J., Olman, V. and Xu, Y. (2009) DOOR: a database for prokaryotic operons. *Nucleic acids research*, **37**, D459-463.

7. Brouwer, R.W., Kuipers, O.P. and van Hijum, S.A. (2008) The relative value of operon predictions. *Brief Bioinform*, **9**, 367-375.
